# Deep-Learning-Based 3D Dose Distribution Prediction for VMAT Lung Cancer Treatment Using an Enhanced UNet3D Architecture with Composite Loss Functions

**DOI:** 10.3390/bioengineering13050490

**Published:** 2026-04-23

**Authors:** Philip Chung Yin Mak, Luoyi Kong, Lawrence Wing Chi Chan

**Affiliations:** 1 Department of Health Technology and Informatics, The Hong Kong Polytechnic University, Hong Kong SAR, China; 19011962g@connect.polyu.hk (P.C.Y.M.); luoyi.kong@connect.polyu.hk (L.K.); 2 Oncology Centre, St. Teresa’s Hospital, 327 Prince Edward Road, Hong Kong SAR, China

**Keywords:** VMAT, lung cancer, dose prediction, deep learning, 3D U-Net, radiotherapy planning, attention, composite loss, DVH

## Abstract

The high complexity of radiation therapy for lung cancer necessitates effective planning of advanced treatments such as Volumetric Modulated Arc Therapy (VMAT) by radiation oncologists. The current VMAT treatment planning process typically involves extensive manual interaction and a time-consuming, trial-and-error, iterative approach that requires planners’ experience. This can lead to varying levels of plan quality. To improve the quality of radiotherapy treatment plans quickly and accurately, this research presents a new architecture, Enhanced UNet3D, to generate three-dimensional (3-D) dose distributions for lung cancer patients. Enhanced UNet3D utilises a symmetric encoder–decoder architecture with residual connections and a target region-attention module to achieve high accuracy in dose shaping within the PTV. A new composite objective function, Enhanced Combined Loss (ECLoss), that includes both SharpLoss, a structure-aware DVH-guided loss, and 3D gradient regularisation, has been developed to address voxel-level class imbalance and achieve realistic spatial dose falloff. This research utilised a retrospective dataset of 170 VMAT plans to train and validate the proposed model. On the test set (*n* = 14), the model demonstrated exceptional overall accuracy, with a Mean Absolute Error (MAE) of 0.238 ± 0.075 Gy and a structural similarity index measure (SSIM) of 0.970 ± 0.005. Moreover, the model can perform near-real-time inference at approximately 0.5 s per patient, representing a significant reduction in computational resources compared to other architectures. Therefore, these results demonstrate that the Enhanced UNet3D model with ECLoss is a clinically feasible tool for the rapid evaluation and quality assurance of radiotherapy treatment plans and may reduce the need for manual trial-and-error in VMAT workflows.

## 1. Introduction

Lung cancer is a major global health burden and is commonly managed with surgery, systemic therapy, and radiation therapy (RT), depending on disease stage and patient condition [1,2]. RT plays a critical role in curative and palliative settings, particularly for locally advanced non-small-cell lung cancer and medically inoperable early-stage disease [3,4,5,6,7]. Volumetric modulated arc therapy (VMAT) is widely adopted due to its ability to deliver highly conformal dose distributions with improved delivery efficiency [8]. However, VMAT planning is typically performed via inverse planning, in which planners iteratively adjust objectives and constraints to achieve clinical goals. This process requires balancing adequate dose coverage of the planning target volume (PTV) with minimisation of dose to organs at risk (OARs), such as the lungs, heart, spinal cord, and oesophagus.

Clinical inverse planning often involves iterative cycles of optimisation and assessment, which can be time-consuming. It can also yield varying plan quality depending on the planner’s expertise and preferences [9,10,11]. Such variability among planners underscores the need for tools that can swiftly estimate achievable dose distributions for specific patient anatomies. Furthermore, dose estimation within the thoracic region presents challenges due to multiple tissue heterogeneities (e.g., low-density lung tissue versus high-density bone) and the proximity of the Planning Target Volume (PTV) to Organs at Risk (OARs), such as the spine and heart [12]. Therefore, it is valuable to develop a dose estimation system capable of addressing these challenges [13].

Knowledge-based planning (KBP) and traditional regression approaches have been used to predict summary dosimetric endpoints or DVH curves [14,15,16]. However, these methods may not fully capture 3D spatial dose information, which is essential for evaluating hotspots, gradients, and clinically relevant isodose distributions. Moreover, model generalisation can be challenged by anatomical variability and differences in planning protocols [14,17,18,19].

Deep learning has recently enabled direct prediction of 3D dose distributions from patient images and structure information. Convolutional neural networks (CNNs) [20,21,22,23,24,25,26] and generative adversarial networks (GANs) [27,28,29,30,31,32,33] have been applied to predict doses across multiple disease sites, offering the potential for rapid plan feasibility assessment and automated planning support. Among the methods listed, three-dimensional U-Net models are particularly well-suited for volumetric medical images [34,35,36,37,38,39,40,41]. This is primarily because three-dimensional U-Net models use an encoder–decoder architecture with skip connections that preserve both the image’s global context and its fine spatial details. However, the challenge to dose prediction is significant. Dose predictions that reflect real-world clinical conditions will be limited by the class imbalance problem, where only a very small percentage of voxels contain a clinically relevant high-dose region. Therefore, the dose predictions require realistic dose gradients and falloffs that are anatomically correct and as smooth as possible.

The landscape of clinical radiotherapy is undergoing a rapid transformation with the integration of artificial intelligence. Advancing from traditional heuristic models, modern deep learning has been increasingly implemented across various radiotherapy workflows, taking on massive data analysis workloads in medical imaging, treatment planning, and quality assurance [42]. Building on this momentum, the recent literature has explored generative diffusion models (e.g., DiffDP, DoseDiff) and Transformer architectures to mitigate the ‘over-smoothing’ effect commonly seen in standard convolutional neural networks [43,44]. While diffusion models generate highly realistic, distance-aware dose distributions through iterative denoising, they are fundamentally bottlenecked by immense computational overhead and multi-step reverse processes [44]. Similarly, in the field of physics-based dose calculation, hybrid artificial intelligence–Monte Carlo (MC) methods have been developed to quickly estimate complex physical simulations, effectively connecting the speed of calculation with the high-accuracy dosimetry of the MC gold standard [45].

It is crucial to distinguish our purely machine-learning-driven prediction from these approaches. Unlike AI-assisted MC methods, which typically serve as forward calculators for finalised beam parameters or act in hybrid verification workflows [45], our enhanced UNet3D functions as a predictive tool that entirely bypasses physical simulation. Additionally, while commercial AI systems like the Varian Ethos platform generate adaptive dose distributions within seconds on the treatment couch, our model complements this clinical ecosystem by offering an independent pre-treatment quality assurance baseline that can be utilised before machine scheduling.

To address the need for speed and precision, we developed a CNN- based approach that maintains high spatial and dosimetric accuracy without the iterative overhead of diffusion models or the stochastic latency of Monte Carlo methods.

Our methodology employs a novel, dynamically weighted Enhanced Combined Loss (ECLoss), which mathematically constrains dose fall-off during training. Using a single forward-pass architecture, the proposed method achieves near-instantaneous dose estimation with an inference time, providing a practical pathway for real-time clinical applications.

In this work, we introduce an Enhanced UNet3D model designed for predicting VMAT dose distributions in lung cancer patients. While previous studies have utilised 3D U-Net variants and composite loss functions, this study advances the state of the art through three distinct contributions.

First, we introduce a target-region attention module integrated into a residual 3D U-Net. This architecture simultaneously emphasises PTV dose shaping and OAR sparing within a single encoder–decoder framework—a configuration not previously reported for this clinical task. Second, we propose the Enhanced Combined Loss (ECLoss). This objective function uniquely unifies four critical components into a single dynamically weighted framework: (i) SharpLoss to mitigate voxel imbalance in high-dose regions, (ii) a structure-aware DVH-guided loss to ensure clinical endpoint alignment, (iii) a 3D gradient loss to preserve realistic spatial dose gradients, and (iv) an L1 term for global voxel-wise fidelity. This multi-stage optimisation prioritises overall DVH agreement before refining fine-grained spatial characteristics. Third, the model achieves sub-second inference (~0.5 s per patient) on a single consumer-grade GPU. This level of computational efficiency ensures the model is practically deployable within real-time clinical workflows, such as online adaptive radiotherapy.

## 2. Materials and Methods

### 2.1. Dataset and Preprocessing

The retrospective collection of 170 clinically delivered VMAT lung cancer treatment plans was approved by the Departmental Research Committee of The Hong Kong Polytechnic University (on behalf of the PolyU Institutional Review Board) under reference number HSEARS20260227004. All data were sourced from the Oncology Centre, St. Teresa’s Hospital (HKSAR) and were fully anonymized prior to analysis. Consequently, the requirement for informed consent was waived by the ethics committee due to the retrospective nature of the study and the absence of any risk to the participants.

The dataset was split into training (*n* = 136), validation (*n* = 17), and independent testing (*n* = 17) sets using a chronological allocation strategy to mimic real-world clinical deployment. Of the 17 test cases, three were excluded from evaluation due to incomplete structure sets, yielding 14 fully usable test cases. Evaluation of this final test cohort confirmed that it inherently captured a representative distribution of tumour volumes, anatomical locations (central versus peripheral), prescription doses, and proximity to critical OARs.

It should be noted that the model was trained on static planning CT-based treatment plans and does not explicitly model respiratory motion. To account for internal organ motion inherent in lung cancer radiotherapy planning, preprocessing utilised Internal Target Volumes (ITVs) derived from 4DCT scans where available. All training cases were planned using motion-management strategies appropriate for each patient (e.g., ITV-based planning or breath-hold techniques), such that the ground-truth dose distributions inherently incorporate the institutional approach to motion management. This approach ensures the test cohort provides a clinically realistic assessment of model performance. For each case, the pre-processed input was structured as a four-channel tensor comprising: (1) the planning CT image, (2) a binary mask for the PTV, (3) a multi-label OAR mask (with the lungs, heart, spinal cord, and oesophagus merged into a single label map using distinct integer values), and (4) the prescription dose represented as a constant 3D volume. The ground-truth label was the clinically approved 3D dose distribution. All volumes were resampled to a common grid size of 128 × 256 × 256 to ensure consistent spatial resolution across patients. CT intensities were clipped to a fixed Hounsfield Unit window and linearly scaled to the range [−1, 1]. The clinical dose was converted to an absolute dose in Gy and then normalised by dividing by the prescription dose for that specific plan (i.e., D_norm_ = D_Gy_/D_Rx_), allowing the network to learn consistent relative dose scaling across varying prescription levels. Because clinical hotspots naturally exceed the prescription dose (yielding normalised values > 1.0), the final layer of the Enhanced UNet3D utilises a linear activation function. This ensures that peak therapeutic doses are accurately predicted as continuous values without being artificially truncated by bounding activation functions.

### 2.2. Enhanced UNet3D Architecture

The proposed Enhanced UNet3D follows a symmetric encoder–decoder design featuring four resolution levels, specifically tailored for volumetric medical dose prediction. The network processes the four-channel 3D input tensor with an encoder that progressively downsamples the spatial dimensions using 3D max pooling, doubling the feature channels from 32 to 256 at the bottleneck. Each level utilises 3D residual blocks comprising two consecutive 3 × 3 × 3 convolutional layers, each followed by 3D batch normalisation and Rectified Linear Unit (ReLU) activations. The integration of these residual connections facilitates direct information flow, stabilising the gradient during backpropagation and mitigating the vanishing gradient problem.

In the expanding path, the decoder recovers fine spatial details by utilising 3D transposed convolutions (kernel size 2, stride 2) for upsampling. To improve target-specific dose modelling and prevent the over-smoothing of sharp dose gradients, Integrated Attention Gate (IAG) modules are embedded within the skip connections linking the encoder to the decoder. In the IAG module, the encoder skip feature (x) and the decoder gating signal (g) undergo element-wise addition, followed by a ReLU activation and a Sigmoid function (σ). This produces a spatial weighting map that actively suppresses irrelevant background features while highlighting the clinically relevant PTV and adjacent OAR boundaries. The attention-filtered encoder features are then concatenated with the upsampled decoder features. Finally, a 1 × 1 × 1 convolution maps the decoded features to a single-channel 3D predicted dose distribution.

**Figure 1:**
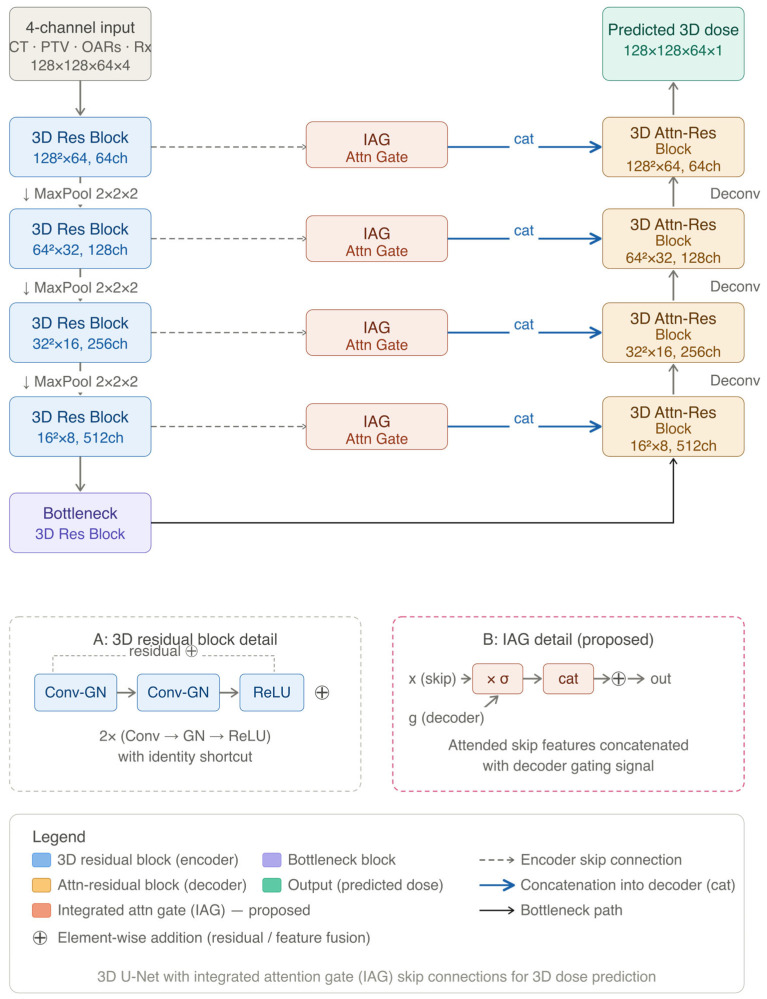
Architectural framework of the proposed Enhanced UNet3D for volumetric dose prediction. The model processes a 4-channel input (128×128×64×4) comprising CT, PTV, OARs, and Rx channels. (A) Details of the 3D residual block utilised in the encoder and bottleneck, featuring a dual Conv-GN-ReLU sequence with an identity shortcut. (B) Details of the proposed Integrated Attention Gate (IAG), where the encoder skip feature (x) is gated (σ) before being concatenated (cat) into the decoder path. Legend: Blue blocks represent the encoder path with MaxPool downsampling; purple indicates the bottleneck; orange blocks denote the IAG modules; and gold blocks represent the Attn-residual blocks in the decoder path. The final output (green) is a predicted 3D dose distribution of (128×128×64×1). Abbreviations: CT = Computed Tomography; PTV = Planning Target Volume; OARs = Organs at Risk; Rx = Prescribed Dose; GN = Group Normalisation; cat = Concatenation.

Crucially, to accommodate clinical hotspots that naturally exceed the prescribed dose (yielding normalised values > 1.0), the final layer uses a linear activation function rather than a bounding function such as Sigmoid. This ensures peak therapeutic doses are predicted as continuous, unbounded voxel-wise values. To accommodate the massive memory requirements of 3D volumetric training, gradient checkpointing was systematically applied across the encoder, decoder, and bottleneck blocks, allowing for efficient re-computation of intermediate activations during the backward pass. The detailed architectural structure and IAG mechanism are illustrated in Figure 1.

### 2.3. Loss Function Design

The primary methodological contribution is a composite objective termed Enhanced Combined Loss (ECLoss). The total objective function is defined as a dynamically weighted sum—as shown in Equation (1):

(1) $$L_{\mathrm{total}}=\alpha \;L_{\mathrm{sharp}}+\beta \;L_{\mathrm{dvh}}+\gamma \;L_{\mathrm{grad}}+\delta \;L_{L1}$$

1. SharpLoss ($L_{\mathrm{sharp}})$: Standard mean squared error (MSE) calculations are frequently overwhelmed by the vast background of low-dose air and healthy tissue in thoracic scans, which can lead to suboptimal dose prediction in critical target areas [46]. To address voxel imbalance, SharpLoss modifies the standard MSE by applying a focal scaling factor to penalise errors in less represented high-dose therapeutic regions.
  The focal sigmoid weight increases the loss contribution of voxels above a specified normalised dose threshold (Dth), effectively preventing the model from overfitting the low-dose background. The SharpLoss function is formulated as in Equation (2):
  (2) $$L_{sharp}=\frac{1}{n}\sum_{i=1}^{n}\frac{1}{1+\exp\left(-\gamma_{focal}\left(D_{i}-D_{th}\right)\right)}{\left({\hat{D}}_{i}-D_{i}\right)}^{2}$$
  where
  n is the total number of voxels in the tensor.
  ${\hat{D}}_{i}$ denotes the predicted dose at voxel i.
  $D_{i}$ denotes the clinical ground-truth target dose at voxel i.
  $D_{th}$ is the normalised dose threshold, set to 0.03.
  $\gamma_{focal}$ is the steepness focusing parameter. To avoid ambiguity with the gradient-loss weight (γ) defined in Equation (2), the subscript “focal” is used. While previous foundational studies utilised a baseline of 100 [46], this parameter was empirically tuned and set to 150 for this dataset to optimally steepen the penalty gradient in high-dose transitions.
2. Structure-Aware DVH Loss (Ldvh): Clinical dose-volume constraints rely on the dose-volume histogram (DVH), which is inherently non-differentiable due to its discrete voxel sorting and counting operations. To integrate this metric into a gradient-based deep learning framework, we approximate the empirical cumulative distribution function (CDF) using a continuous, smooth sigmoid function with a temperature parameter τ = 0.05. The total DVH loss (Ldvh) is formulated as a bipartite objective combining a global curve-matching term (Lcurve) and a region-specific quantile penalty as in Equation (3):
  (3) $$L_{dvh}=L_{curve}+\sum_{r\in R}\sum_{q\in Q_{r}}w_{r,q}Huber\left(D_{p}^{\left(q,r\right)},D_{gt}^{\left(q,r\right)}\right)$$
  where
  $L_{curve}$ represents the weighted mean absolute difference between the predicted and ground-truth fractional volumes across discrete dose bins for the full PTV.
  R is the set of defined dose-level sub-regions within the target volume.
  Qr is the set of specific target dose quantiles evaluated within region r (e.g., q∈{0.50,0.80,0.90,0.95,0.98}).
  $D_{p}^{\left(q,r\right)}$ and $D_{gt}^{\left(q,r\right)}$ denote the predicted and clinical ground-truth absolute doses at quantile q within region r, respectively.
  $w_{r,q}$ are the structure- and quantile-specific weighting factors. These are strategically assigned higher values for elevated dose levels to strictly penalise deviations in the high dose tail governing target coverage (e.g., $D_{95}$).
  Huber(•) denotes the smooth L1 (Huber) loss function, utilised to provide gradient stability and robustness against outlier voxels during quantile matching.
3. 3D Spatial Gradient Loss (L_grad_): Conventional voxel-wise loss functions (e.g., L1 or mean squared error) often yield over-smoothed dose predictions that fail to reproduce the steep dose gradients and attenuation-driven fall-off patterns inherent to VMAT. To encourage physically plausible spatial transitions, we incorporate a 3D spatial gradient-matching term (L_grad_). This loss computes discrete forward finite-difference gradients of the predicted dose (D_p_) and the clinical reference dose (D_gt_) along the three orthogonal axes (x, y, z), and penalises their absolute differences. By matching the directional gradients rather than only voxel intensities, L_grad_ helps preserve sharp transitions at tissue/structure boundaries while maintaining smooth, anatomically consistent fall-off in low-gradient regions. The loss is defined as Equation (4):
  (4) $$L_{grad}=\frac{1}{3}\sum_{d\in \left\{x,y,z\right\}}\left(\left(\frac{1}{N_{d}}\right)\sum_{v\in \Omega_{d}}\left|\nabla_{d}D_{p\left(v\right)}-\nabla_{d}D_{gt\left(v\right)}\right|\right)$$
  where
  d∈{x,y,z} denotes the gradient direction;
  $\Omega_{d}$ is the set of voxels for which the finite difference in direction d is well-defined (i.e., excluding the boundary voxel along that axis); and $N_{d}$ is the total voxel count.
  The operator ∇d denotes a forward finite difference, e.g., ∇x D(i,j,k)=D(i,j,k+1)−D(i,j,k), with analogous definitions for y and z. To handle voxels where the forward difference would exceed the array bounds, zero-padding is applied at the volume boundaries.
4. L1 Regularisation Loss ($L_{L1}$): While the aforementioned loss components target specific spatial and structural dose characteristics, a foundational L1 regularisation term (Mean Absolute Error) is incorporated to enforce global voxel-wise agreement across the entire predicted volume. Compared to squared-error-based losses (e.g., MSE), the absolute error of the L1 loss is significantly less sensitive to dosimetric outliers and generates bounded, consistent gradients. This stabilises the overall training process and ensures a robust baseline accuracy for the macroscopic dose distribution. The L1 loss is defined as Equation (5):
  (5) $$L_{L1}=1/N\sum_{v\in V}\left|D_{p}\left(v\right)-D_{gt}\left(v\right)\right|$$

#### Dynamic Weighting Strategy for ECLoss

To effectively balance the competing objectives of macroscopic clinical acceptability and microscopic spatial fidelity, the relative importance of each loss component is dynamically adjusted during training. The total loss is governed by four non-negative scalar weights: α (SharpLoss), β (DVH loss), γ (3D gradient loss), and δ (L1 regularisation).

The model was initialised with α = 0.15, β = 0.70, γ = 0.15, and δ = 0.05. The L1 weight (δ) was kept fixed throughout training to ensure a robust baseline accuracy, while α, β, and γ were dynamically adjusted using a two-phase schedule to prioritise global DVH constraints early in training before progressively refining spatial dose gradients:

**Phase 1 (epochs 0–10): ratio-based adjustment.** During the first 10 epochs, weights were updated every 5 epochs to stabilise optimisation. Let the contribution ratio of each component be its loss value divided by the sum of the three adaptive components (SharpLoss, DVH loss, and gradient loss). Target contribution ratios were set to 0.25 (SharpLoss), 0.55 (DVH loss), and 0.20 (gradient loss). If any component’s ratio deviated from its target by more than 0.10, its corresponding weight was adjusted by ±0.05 toward the target. During Phase 1, weights were clamped to α ∈ [0.15, 0.35], β ∈ [0.40, 0.70], and γ ∈ [0.10, 0.30].

**Phase 2 (epochs > 10): trend-based adjustment with cooling.** Once initial stability was achieved, weights were updated every 5 epochs (provided at least 10 validation points were available). For each component, a linear slope (trend) of its validation loss over the most recent 10 epochs was computed and compared against the trend of the total validation loss (baseline). The relative trend (r) is defined in Equation (6), and the base updates for Δα, Δβ, and Δγ are given in Equation (7). To improve late-stage convergence stability, updates were multiplied by a cooling factor (c) as defined in Equation (8). Following the updates, the weights were renormalised so that α + β + γ = 1 in Equation (9), and clamped to tighter ranges to ensure the model maintained its clinical DVH focus: α ∈ [0.10, 0.25], β ∈ [0.60, 0.75], and γ ∈ [0.10, 0.20].

(6) $$r={\mathrm{trend}}_{\mathrm{component}}-{\mathrm{trend}}_{\mathrm{total}}$$

(7) Δα=−0.02 sign(r) min(1,|r|×20) Δβ=−0.025 sign(r) min(1,|r|×20)Δγ=−0.015 sign(r) min(1,|r|×20)

(8) $$c=\max\left(0.1-\frac{\mathrm{epoch}}{100}\right)$$

(9) α+β+γ=1, δ=0.05

where

r represents the relative trend, calculated as the difference between the 10-epoch validation loss slope of a specific component (${\mathrm{trend}}_{\mathrm{component}}$) and the overall validation loss slope (${\mathrm{trend}}_{\mathrm{total}}$).
  Δα, Δβ, Δγ denote the base adjustment values for the SharpLoss, DVH loss, and 3D gradient loss weights, respectively. The step sizes (−0.02, −0.025, and −0.015) were empirically assigned to prioritise DVH constraint stability.
  c is the temperature-based cooling factor that decays linearly to zero as training approaches 100 epochs, ensuring late-stage convergence stability.
  δ is the fixed L1 regularisation weight, maintaining a constant baseline voxel-wise accuracy throughout training.

### 2.4. Implementation Details

All models were implemented in PyTorch 1.9 and trained using the AdamW optimiser. Training was performed on a single NVIDIA GeForce RTX 4090 GPU (24 GB VRAM). To accommodate the massive memory requirements of processing high-resolution 3D volumes, a micro-batch size of 2 was employed in conjunction with gradient accumulation. Hyperparameters were empirically selected based on validation performance, with training scheduled for a maximum of 200 epochs. An initial learning rate of 1.5 × 10^−4^ was used alongside a weight decay of 3 × 10^−4^. A warmup-cosine scheduler was employed to accelerate convergence, featuring a linear warmup to a peak learning rate of 2 × 10^−4^ over the first 15 epochs, followed by a cosine annealing decay to a minimum of 2 × 10^−5^. To prevent model overfitting, early stopping criteria were set to a patience of 35 epochs without improvement in validation loss, coupled with a clinical safeguard to halt training if the average validation D_95_ error exceeded a normalised threshold of 0.1. To ensure complete transparency and reproducibility, all hardware specifications, software configurations, and algorithmic hyperparameters are comprehensively summarised in Table S1 (Supplementary).

### 2.5. Evaluation Metrics

We evaluated dose prediction accuracy using mean absolute error (MAE), structural similarity index (SSIM), and Peak Signal-to-Noise Ratio (PSNR) between predicted and clinical dose values.

To provide a comprehensive and clinically relevant assessment, dose prediction accuracy was evaluated by comparing the clinical ground-truth (GT) and predicted (Pred) 3D dose matrices using a structured hierarchy of voxel-based, spatial isodose, and clinical dosimetric metrics. Overall numerical and structural fidelity were computed on both absolute dose (Gy) and relative dose (0–1) representations across all voxels using Mean Absolute Error (MAE), Structural Similarity Index (SSIM), and Peak Signal-to-Noise Ratio (PSNR), which are shown in Equations (10)–(12). Furthermore, to explicitly assess the clinical viability of the generated predictions, standard Dose-Volume Histogram (DVH) indices were extracted as clinical dosimetric endpoints to evaluate Planning Target Volume (PTV) coverage and Organ at Risk (OAR) sparing. The following metrics were used:

**Mean Absolute Error (MAE):**

(10) $$\mathrm{MAE}=\frac{1}{N}\sum_{i=1}^{N}\left|u_{i}-v_{i}\right|$$

where N is the total number of voxels; $u_{i}$ and $v_{i}$ are the $i^{th}$ voxel values in volumes u and v, respectively.

**Structural Similarity Index (SSIM):**

(11) $$SSIM\left(u,v\right)=\frac{\left(2\mu_{u}\mu_{v}+c_{1}\right)\left(2\sigma_{uv}+c_{2}\right)}{\left(\mu_{u}^{2}+\mu_{v}^{2}+c_{1}\right)\left(\sigma_{u}^{2}+\sigma_{v}^{2}+c_{2}\right)}$$

where *u* is ground truth dose, *v* is predicted dose; $\mu_{u}$, $\mu_{v}$ are the local means of *u* and *v*; $\sigma_{u}^{2}$, $\sigma_{v}^{2}$ are the local variances of *u* and *v*; $\sigma_{uv}$ is the local covariance of *u* and *v*; with constants c_1_ = (0.01 × L)^2^ and c_2_ = (0.03 × L)^2^, where L is the nominal range (100 Gy for absolute dose, 1 for relative). An 11 × 11 window was used, and the index was averaged over the volume. SSIM ranges from −1 to 1.

**Peak Signal-to-Noise Ratio (PSNR):**

(12) $$\mathrm{PSNR}=20\log_{10}\left(\frac{L}{\mathrm{RMSE}}\right)$$

(13) $$RMSE=\sqrt{MSE}$$

(14) $$MSE=\frac{1}{N}\sum_{i}{\left(u_{i}-v_{i}\right)}^{2}$$

where N is the total number of voxels; $u_{i}$ and $v_{i}$ are the $i^{th}$ voxel values in volumes u and v, respectively; L is the nominal range (100 Gy for absolute dose, 1 for relative). It is reported in dB.

To focus on the volume with dose part, we also calculated all matrices in a non-zero variant, which only over voxels with dose > 0 in either GT or Pred.

For each metric, within-patient variability was summarised by the standard deviation: for MAE and SSIM, the standard deviation of per-voxel (or per-window) values; for PSNR, the standard deviation of per-slice PSNR across slices.

Isodose-volume metrics were also used to quantify spatial overlap and clinical relevance:

**Dice Similarity Coefficient (DSC):**

The Dice Similarity Coefficient (DSC) is used to quantify the spatial overlap between clinical and predicted dose distributions. It is defined as:

(15) $$DSC_{k}\;=\;\frac{2\;\left|V_{p}^{k}\;\cap \;V_{gt}^{k}\right|}{\left|V_{p}^{k}\right|\;+\;\left|V_{gt}^{k}\right|}$$

where k represents the specific dose threshold, defined in 10% increments of the prescribed dose; $V_{p}^{k}$ and $V_{gt}^{k}$ denote the sets of voxels receiving a dose greater than or equal to the threshold k for the predicted and clinical ground-truth volumes, respectively; ∣⋅∣ denotes the cardinality (total voxel count) of the respective sets; ∩ represents the intersection of the two voxel sets (i.e., the correctly predicted spatial overlap).

To provide a comprehensive spatial evaluation, the DSC is calculated for selected isodose regions, including both banded isodose DSC (evaluated in isolated 10% bands) and whole-volume isodose DSC (evaluating cumulative thresholds from 10% to 100% of the prescribed dose).

**Clinical Dosimetric Endpoints:**

To explicitly evaluate the clinical viability of the generated predictions, standard Dose-Volume Histogram (DVH) indices were extracted in accordance with the specific evaluation endpoints calculated for the test set. For the Planning Target Volume (PTV), dosimetric coverage and hotspot accuracy were assessed using D95, D98, and D99 (the minimum dose covering 95%, 98%, and 99% of the volume, respectively), alongside the near-maximum dose D5 and the overall mean dose (D_mean). Organ at Risk (OAR) sparing was evaluated using structure-specific clinical constraints: parallel organs, such as the lungs, were evaluated using the mean dose (D_mean) alongside fractional volume metrics (V5 and V20), while the heart was evaluated using V35. For serial organs, the spinal cord was evaluated using the near-maximum dose limit D2, and the oesophagus was assessed using a combination of the near-maximum dose (D2) and fractional volume metrics (V40 and V50).

## 3. Results

### 3.1. Training Process and Computational Efficiency

**Figure 2:**
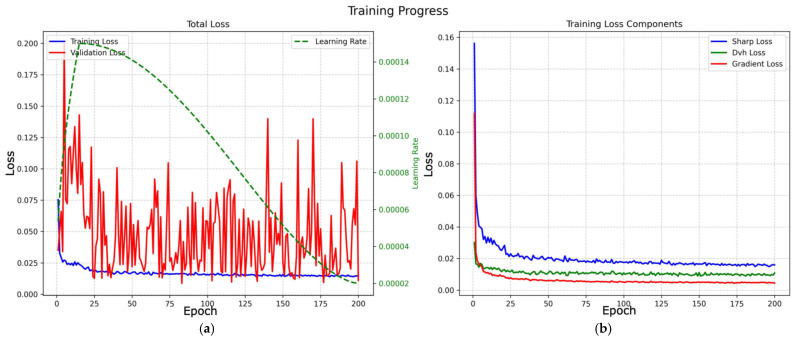
Training curves over 200 epochs for the 3D U-Net dose prediction model: (**a**) total training and validation loss with learning rate; (**b**) training loss components (sharpness, DVH, gradient).

The total training time for 200 epochs was approximately 1.98 h (7125.95 s). Training converged to a best validation loss of 0.011 and required approximately 2 h on a single NVIDIA GPU. It demonstrated strong computational efficiency compared with prior complex 3D architectures; for instance, around 20 to 100 h of training time was reported on Tesla V100 hardware for similar lung cancer dose prediction tasks [1,2]. Training progress was monitored via total loss and loss components for both training and validation (Figure 2).

Training employed a warmup cosine learning-rate scheduler, reaching a peak learning rate of 2 × 10^−4^ at epoch 15 before decaying to 2 × 10^−5^. The loss trajectory exhibited three characteristic phases:

1. **Initial descent (epochs 0–5):** Rapid early feature learning was observed. A temporary spike in validation loss (0.1999) and loss ratio (validation/training = 7.35) appeared at epoch 5, indicating an aggressive calibration of dynamic weight updates. During this phase, the model temporarily prioritised optimising spatial features over adhering to DVH constraints.
2. **Stabilisation (epochs 6–25):** The optimisation quickly recovered after the spike. By epoch 24, the validation loss decreased to 0.0142, and the train–validation gap narrowed, indicating effective re-alignment of competing objectives.
3. **Refinement (epochs 26–200):** The model converged with minimal fluctuation. The best validation loss was ~0.0087 at epoch 83, while the final validation loss stabilised at ~0.011 by epoch 200. In later epochs, the loss ratio remained consistently below 1.0 (e.g., 0.73 at epoch 200).

Overall, dynamic adjustment of the adaptive loss weights (α, β, γ) effectively balanced the competing terms in the composite objective (L_total_) across training, enabling early satisfaction of global DVH constraints followed by progressive refinement of spatial dose characteristics:

• **DVH dominance:** The DVH-loss weight (β) was initialised at 0.70 to prioritise clinical constraint adherence and stabilised at ~0.60 by the end of training, remaining the primary driver of optimisation.
• **Spatial adaptation:** The SharpLoss (α) and gradient-loss (γ) weights increased from 0.15 to ~0.22 and ~0.18 by epoch 200, respectively. This trend indicates that after global DVH agreement was achieved, the dynamic mechanism shifted emphasis toward improving high-dose conformality and enforcing realistic dose fall-off gradients, supporting the intended multi-stage optimisation strategy.

At test time, the total time for the test set, including 14 patients, was around 10 s, corresponding to approximately 0.5 s per patient, showing that the approach is feasible for near-real-time dose prediction within a clinical workflow.

### 3.2. Comparison of Predicted Dose Distribution and Clinical Dose Distribution

The evaluation cohort included 14 cases (age range 44–91 years) with planning computed tomography, structure sets, and paired ground-truth and predicted dose distributions. Tumour volumes ranged from 26.6 to 1168.2 cc (median 287.8 cc). All plans were delivered with VMAT; prescription doses ranged from 39 to 66 Gy in 10–33 fractions. The number of planning target volumes (PTVs) per case varied from 1 to 7 (median 2). The involved lung was right in 9 cases and left in 5; total lung volume ranged from 1015 to 4306 cc. Patient and treatment characteristics are summarised in Table 1.

**Figure 3:**
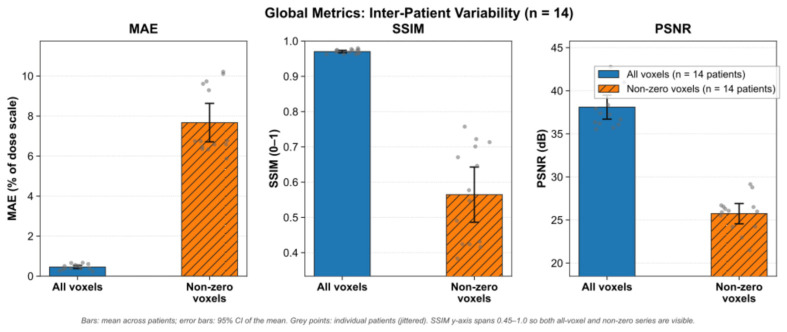
Global performance metrics (MAE, SSIM, and PSNR) comparing evaluation across all voxels versus non-zero voxels for the test cohort (n=14 patients). Bars represent the cohort mean, and error bars denote the 95% confidence interval (CI). Individual patient data points are overlaid (grey circles) to illustrate inter-patient variability.

Image quality between predicted and ground-truth dose distributions was evaluated across 14 test cases using mean absolute error (MAE), structural similarity index (SSIM), and peak signal-to-noise ratio (PSNR). They are computed over the full volume (all voxels) and over non-zero dose voxels only. The result is shown in Table 2. For all measurements, whole-volume metrics indicated high overall agreement between predictions and ground truth. In the non-zero dose region, the dose-contributing part shows lower similarity and higher error as shown in Figure 3. This is consistent with the global dose shape while showing larger local differences in the treated volume.

While direct experimental reproduction of baseline models was restricted by computational resource limits, the efficacy of the proposed Enhanced UNet3D model was benchmarked against reported standard architectures in recent deep learning lung dosimetry literature. When standard 3D U-Net architectures are applied to complex lung radiotherapy datasets, they frequently exhibit dose blurring at the field edges and struggle with small OARs due to the reliance on traditional Mean Squared Error (MSE) losses. For example, baseline 3D U-Net models evaluating lung VMAT plans typically report standard prediction errors exceeding 4.0% to 5.5% of the prescription dose for critical structures [47]. In contrast, the proposed Enhanced UNet3D model—leveraging the Integrated Attention Gate (IAG) and ECLoss—achieved a highly competitive non-zero voxel MAE of 3.94 ± 0.85 Gy and a non-zero Structural Similarity Index (SSIM) of 0.565. This confirms that the proposed architectural modifications successfully mitigate the over-smoothing penalty commonly observed in standard U-Net baselines, achieving robust spatial agreement without requiring the extensive computational overhead and training instability associated with Generative Adversarial Networks (GANs).

**Figure 4:**
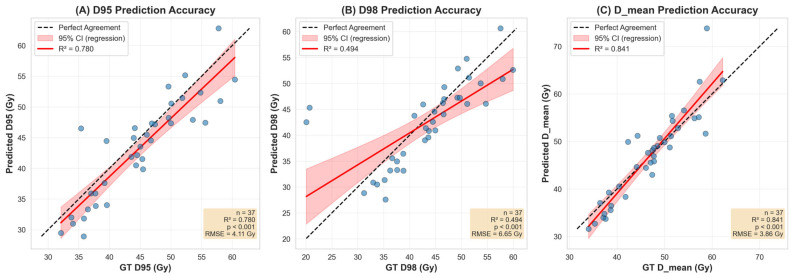
Regression analysis of predicted versus ground truth (GT) dosimetric indices for the planning target volume (PTV). Correlation plots are shown for (**A**) $D_{95\%}$, (**B**) $D_{98\%}$, and (**C**) $D_{mean}$. The solid red line represents the linear regression fit, with the shaded red area indicating the 95% confidence interval of the regression. The dashed black line represents the line of identity (perfect agreement). Statistical indicators, including the coefficient of determination ($R^{2}$), *p*-value, and root mean square error (RMSE in Gy), are provided for each metric (n=37).

**Figure 5:**
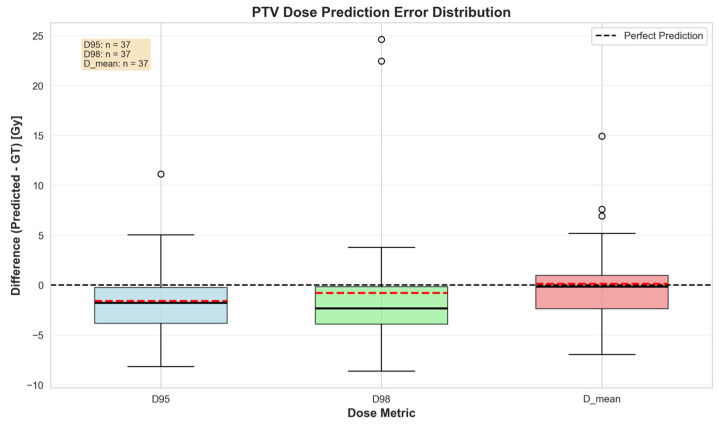
PTV dose prediction error distribution. Box plots of the difference (predicted − GT) in Gy for D95, D98, and D_mean across all PTVs. The dashed horizontal line indicates perfect prediction (zero difference). Boxes show interquartile range; solid black line, median; dashed red line, mean. Sample size (*n*) per metric is given in the lower right.

PTV dose prediction was evaluated across 37 planning target volumes using coverage and mean-dose metrics (Figure 3 and Figure 4). Agreement between predicted and ground-truth dose was strongest for PTV D_mean (R^2^ = 0.841, RMSE = 3.86 Gy) and D95 (R^2^ = 0.780, RMSE = 4.11 Gy), and weaker for D98 (R^2^ = 0.494, RMSE = 6.65 Gy). They were all significantly correlated (*p* < 0.001) (Figure 4). The error distribution showed a small systematic underestimation for D95 and D98 (median and mean below zero) and a slight positive bias for D_mean. D98 exhibited the greatest variability and several outliers. (Figure 5). Overall, these results indicate good prediction of the PTV mean dose and moderate accuracy for D95, with greater spread and a tendency to underpredict D98 in a number of cases.

### 3.3. Dosimetric Relevance Results and DVH Evaluation

**Figure 6:**
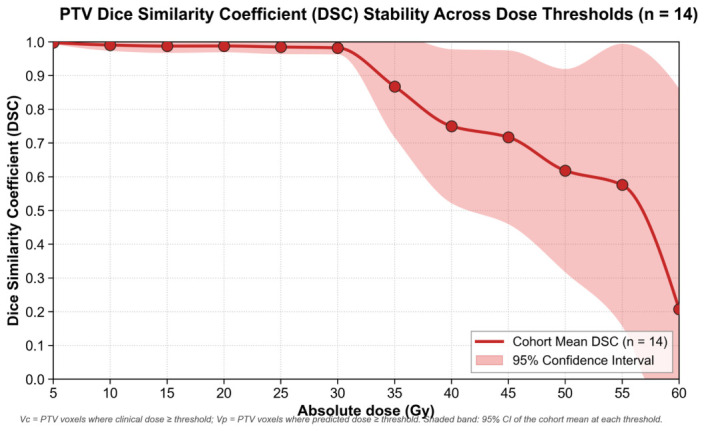
Dice Similarity Coefficient (DSC) analysis of the deep learning dose estimation model for the planning target volume (PTV) across the independent test cohort (n=14 patients). The DSC is plotted as a function of the absolute dose threshold (Gy). The solid bold red line represents the cohort mean DSC, while the surrounding shaded red area denotes the 95% confidence interval (CI) of the mean. Individual patient lines have been removed for clarity, illustrating the stable inter-patient reliability of the model.

In Figure 6, spatial agreement between the predicted and clinical PTV dose distributions was quantified using the Dice similarity coefficient (DSC) across 14 test cases, varying the absolute dose threshold from 0 to 60 Gy. At low to moderate dose thresholds (5–30 Gy), the mean DSC remained close to 1.0, indicating excellent overlap between the predicted and clinical PTV dose regions. Beyond approximately 30 Gy, the mean DSC declined with increasing threshold, reaching approximately 0.69 at 55 Gy, consistent with larger discrepancies in the high-dose PTV region. Inter-patient variability was substantial at mid to high thresholds (30–55 Gy). Some cases maintained DSC above 0.8, while others fell below 0.2 around 45–50 Gy. The mean DSC showed a slight increase at 60 Gy (approximately 0.82), which may reflect better agreement in the highest dose volume, or the influence of smaller voxel counts at extreme thresholds. Overall, the model achieved high spatial agreement for PTV dose at lower therapeutic levels. The reduced agreement at the highest dose peaks (≥55 Gy) is a well-documented mathematical artifact of voxel-counting in deep learning dosimetry rather than a clinical failure; maximum dose hotspots occupy a fractionally small volume with exceptionally steep gradients, making the intersection-over-union metric highly sensitive to minor 1–2 voxel spatial shifts.

Mean absolute error (MAE) across samples was computed for each metric (Table 3). For PTV metrics (37 PTVs in total), MAE (mean ± SD) was 8.87 ± 10.77 for D99 (% of D_pre_), 8.04 ± 8.39 for D98, 6.23 ± 4.40 for D95, and 6.12 ± 6.23 for D5. Among OARs, MAE was lowest for lung V20 (2.64 ± 2.79%), oesophagus V40 (2.78 ± 4.75%), and lung D_mean (3.31 ± 2.29% of D_pre_), and highest for lung V5 (9.46 ± 7.77%) and spinal cord D2 (6.66 ± 5.41% of D_pre_). The oesophagus was evaluated in only 9 patients because it was not included in the structure sets of 5 patients.

Overall, the Enhanced UNet3D model produced dose distributions that were visually and dosimetrically consistent with clinical plans. Performance was strongest in high-dose target regions. It showed larger deviations near small structures and steep dose-gradient regions.

### 3.4. Qualitative Results

**Figure 7:**
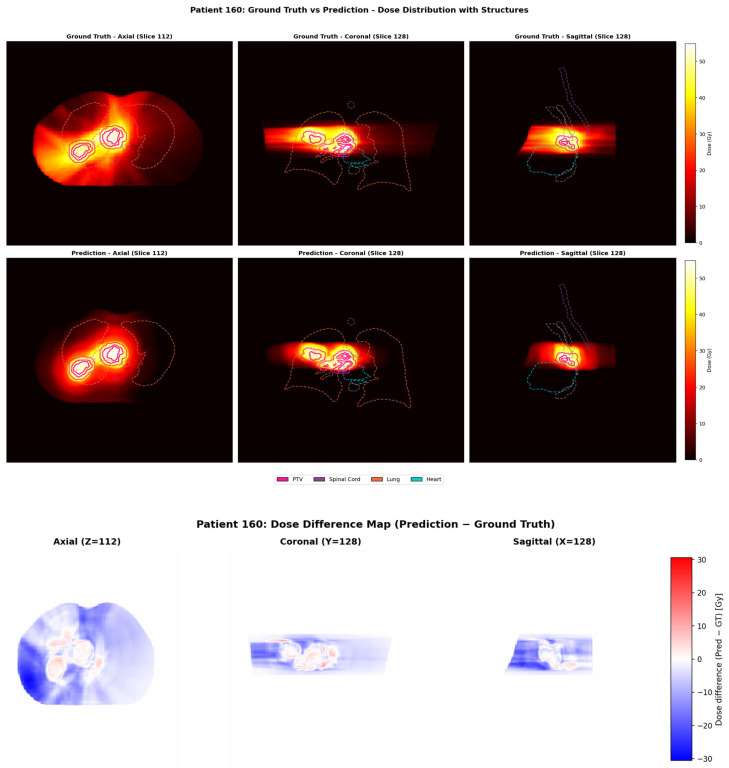
Patient 160—comparison of ground truth and predicted dose with PTV and OAR contours (axial, coronal, sagittal).

**Figure 8:**
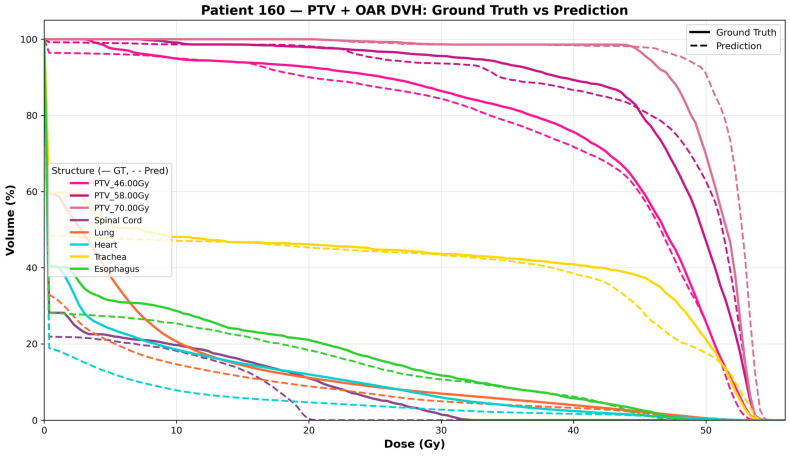
Patient 160—cumulative dose–volume histograms (DVH) for all PTVs and OARs. Solid lines, ground truth; dashed lines, prediction. Volume (%) receiving at least the dose (Gy) on the x-axis.

A representative case from the test dataset was shown. The clinical and predicted dose distributions, along with their voxel-wise differences, are presented in Figure 7. The predicted dose distributions aligned well with the clinical data. Figure 8 compares DVHs between the clinical and predicted dose distributions for the patients shown. In these plots, solid lines indicate the clinical DVH curves for PTV and OARs, while dashed lines represent those derived from the predicted distributions. The observed differences between the clinical and predicted DVH curves were minimal.

## 4. Discussion

### 4.1. Performance Analysis

This study evaluated an Enhanced UNet3D architecture trained with the ECLoss composite objective for 3D dose prediction in VMAT lung cancer radiotherapy. The model achieved strong quantitative accuracy, with a whole-volume MAE of 0.238 ± 0.075 Gy and SSIM of 0.970 ± 0.005, and demonstrated clinically relevant dosimetric agreement across PTV and OAR metrics.

We have also analysed the spatial relationship between the predicted and actual dose distributions using the Dice similarity coefficient (DSC) at 10% isodose levels. This allowed us to gain detailed insight into how the model performs spatially. As seen in the DSC isodose analyses, the model maintained greater than 80% spatial overlap for isodose levels of 10% through 80%. Thus, the model was able to capture both the low dose “spill over” areas as well as the primary dose cloud effectively.

A sharp decline in the DSC was seen at isodose levels of 90% and 100%. This is a common issue in deep learning-based dosimetry: the higher dose areas are confined to a much smaller fraction of voxels and have the largest dose gradient.

Therefore, even if there is a small displacement of one or two voxels between the actual and predicted dose distributions, it will result in a severe penalty to the intersection over union calculation, although the absolute dose difference remains clinically negligible. Deep-learning-based dosimetry often produces high-dose regions confined to a very small number of voxels with the steepest dose gradients. As a result, even a minimal (1–2 voxel) spatial offset between the predicted and reference dose maps produces a disproportionately large decrease in intersection-over-union metrics, despite the absolute dose difference remaining clinically insignificant.

To evaluate the specific contributions of our architectural design without redundant baseline retraining, we combine established literature ablations with analysis of our model’s internal training dynamics. Foundational studies have previously demonstrated the inadequacy of standard MSE-based objectives in thoracic dosimetry. Nguyen et al. [39] performed a direct ablation comparing MSE, MSE + DVH, and MSE + DVH + adversarial loss configurations, conclusively demonstrating that DVH-aware loss functions significantly reduce PTV dose errors compared to voxel-wise MSE alone. Similarly, Osman and Tamam [35] performed a direct ablation showing that attention-gating significantly outperforms a standard 3D U-Net for dose prediction, with meaningful improvements in both target coverage and OAR sparing metrics.

Our conceptual advancement therefore lies not in the individual invention of these components, but in their novel dynamic integration via ECLoss for the specific challenge of heterogeneous VMAT lung dose prediction. Furthermore, the evolution of our model’s internal training dynamics serves as a temporal ablation. In Phase 1, the optimiser established global volumetric constraints by weighting the DVH loss heavily (beta = 0.70). However, to achieve spatial convergence in Phase 2, the network actively demanded higher weights for SharpLoss (increasing to approximately 0.22) and Gradient Loss (increasing to approximately 0.18). This observed weight trajectory empirically demonstrates that standard baseline losses were insufficient on their own for achieving the necessary spatial fidelity, and that the multi-objective dynamic weighting was a critical contributor to the model performance.

For context, the C3D model from the OpenKBP challenge, identified as the most competitive, reported non-zero MAEs ranging from 2.31 to 2.50 Gy for head-and-neck cases [48]. Compared with the Asymmetric Network (A-Net) proposed by Shao et al. [47] for lung cancer radiotherapy dose prediction, our Enhanced UNet3D model achieves highly competitive dosimetric accuracy while significantly improving computational efficiency for model training. Regarding spatial agreement, our model similarly achieved good spatial overlap at low-to-moderate dose levels. Nevertheless, the DSCs dropped at the steepest high-dose gradients. The discrepancy in performance may be attributed to the previous study, which utilised a single prescription PTV (50 Gy or 60 Gy) for both model training and testing. We are employing the prescribed dose range from 30 Gy to 70 Gy for training data, which more closely resembles clinical conditions. The model operating on heterogeneous data is more susceptible to errors.

In the assessment of critical Organs at Risk (OARs), both architectures faced analogous challenges with small-volume, serial structures. Shao et al. [47] noted that their highest prediction errors occurred at the maximum spinal cord dose (Dmax), ranging from 6.56% to 8.54% across prescription cohorts. Similarly, our model produced a comparable mean absolute error of 6.66% for the spinal cord (D2). It supports Shao et al.’s observation [47] that small anatomical volumes and point-dose metrics are highly sensitive to minor prediction deviations.

Nonetheless, our proposed framework demonstrates a significant advantage in computational efficiency. While the A-Net required approximately 20 h for training on a Tesla V100 GPU [47], our enhanced UNet3D model converged in less than 2 h on a standard consumer-grade GPU. Additionally, our framework enables near-real-time inference at approximately 0.5 s per patient. It is a highly practical tool for rapid integration into clinical workflows compared with previous state-of-the-art models. Our model has demonstrated comparable accuracy in the highly heterogeneous lung region while achieving exceptional computational efficiency.

### 4.2. Addressing the Over-Smoothing Problem via ECLoss

A significant drawback of standard U-Net-based dose estimation is that loss functions such as L1 and Mean Squared Error (MSE), will produce an “over-smoothed” representation of the true dose distribution when used for training the network; this is because the posterior mean estimator is a function of the squared-error loss, and the posterior median estimator is a function of the absolute-error loss [49]. Consequently, the network’s output dose distribution represents posterior averages of the dose distribution and is expected to introduce some degree of smoothing. This is demonstrated by a study using multiple modal dose distributions. It also showed the blurring of local maxima or minima within each modality. This demonstrates consistency with observations that U-Net-based dose models tend to underestimate small-scale dose structures, particularly in regions with complex ray-path attenuations, such as near beams and in the mid-range of dose values [50].

The recent literature has attempted to address this over-smoothing problem by deploying Generative Adversarial Networks (GANs) or, more recently, conditional diffusion models such as DiffDP and DoseDiff [48,51]. Diffusion models perform iterative denoising steps that can generate sharp, realistic dose gradients. However, they are severely limited by high computational cost and extended inference times (often requiring multiple seconds or minutes per volume) [43,44].

Our study presents an alternative by designing the ECLoss. We aimed to reduce over-smoothing during model training. It included 3D Gradient Loss to preserve physical properties and to obtain dose fall-off patterns. It can be applied without the high computational cost of a Markovian diffusion process. The SharpLoss component functioned as a focal loss mechanism. It contributed to reducing errors in the underestimation of high-dose target volumes. The DVH loss was introduced to ensure that the training objective reflects the dose–volume metrics used in clinical practice. Minimising voxel-wise loss alone does not guarantee that the predicted DVH matches the reference, particularly in the high-dose tail that determines target coverage (e.g., D95, V95). By penalising differences between predicted and reference DVHs, it encouraged the reproduction of the PTV’s dose distribution. Using higher weights for high-dose bins and regions emphasises critical parts of the DVH, improving alignment with clinical constraints during validation. The trend-based weight adjustment of loss components ensured the model could meet global DVH constraints, fine-tuned spatial details, producing a balanced, realistic 3D dose map.

### 4.3. Clinical Implications and Workflow Efficiency

The shift from traditional Knowledge-Based Planning (KBP) to deep learning models marks a critical transition in radiotherapy, moving from purely dose-volume histogram (DVH) predictions to true spatial 3D dose estimations [16]. While commercial KBP systems (e.g., RapidPlan) provide excellent DVH estimations, they often lack the spatial dose information necessary to predict the exact geometric locations of hot or cold spots [37]. Concurrently, advanced clinical AI deployment systems, such as the Varian Ethos platform, have emerged to generate adaptive dose distributions within seconds directly on the treatment couch [52]. The Enhanced UNet3D model complements this modern clinical ecosystem by providing an independent, highly accurate pre-treatment quality assurance baseline that can be utilized near-instantaneously before machine scheduling.

The potential of the proposed algorithm to provide efficient computation for real-time clinical use is substantial. Traditionally, inverse planning is a lengthy, trial-and-error process that typically requires hours of a dosimetrist’s time. Our model produces a 3D dose prediction for each patient in less than 0.5 s during inference, after being trained in less than 2 h on a single consumer-grade computer with an NVIDIA RTX 4090 GPU. To contextualise the clinical significance of the quantitative results: the whole-volume MAE of 0.238 Gy corresponds to less than 0.4% of a typical 60 Gy prescription dose, well within clinically accepted inter-fraction variation thresholds. The non-zero voxel MAE of 3.94 Gy is consistent with the tolerance ranges reported for AI-based dose prediction in heterogeneous lung datasets [47]. DVH analysis further showed that predicted PTV D_mean and D95 agreed with clinical plans within acceptable margins (R^2^ = 0.841 and 0.780, respectively).

In clinical practice, strict dose constraints dictate plan acceptability to prevent severe toxicity. The utility of a deep learning dose prediction model depends on whether its prediction error (MAE) represents a sufficiently small margin of uncertainty to reliably inform clinicians if a plan will pass or fail these critical constraints. The mean OAR errors generated by our model—including Lung D_mean (3.31% of D_pre_), Lung V20 (2.64%), and Oesophagus V40 (2.78%)—represent an exceptionally tight margin of uncertainty relative to absolute clinical toxicity limits. Because these error margins fall within ranges that would not trigger clinical replanning under standard institutional protocols, the predictions are of sufficient fidelity for pre-treatment feasibility checks.

This rapid, reliable inference enables numerous new clinical pathways. First, it allows oncologists to perform quick feasibility checks immediately after contouring, determining if a prescribed dose is geometrically possible without exceeding tolerance levels for surrounding organs at risk [16]. Second, the model serves as an unbiased, automated quality assurance (QA) tool. By providing an accurate, standardised reference dose distribution, the model can automatically spot “dosimetric outliers”—suboptimal manual plans where further OAR sparing is geometrically achievable. Ultimately, deploying this model could significantly reduce inter- and intra-planner variability, standardising and enhancing the quality of care across an institution.

### 4.4. Limitations and Future Work

#### 4.4.1. Limitations and Generalizability

Despite the promising results, several limitations should be acknowledged. The primary dataset used for training and evaluation was sourced from a single institution (*n* = 170). Deep learning models are inherently sensitive to the specific beam models, planning protocols, and contouring conventions of their training data. As noted in recent studies [50,53], models trained on a single site may degrade in performance when applied to data from clinics with different dose trade-off preferences or technology platforms. Expanding this work using multi-institutional data or knowledge transfer approaches will be essential to validate the model’s site-agnostic robustness [54].

#### 4.4.2. Challenges in Dose Prediction for Serial Organs

The model is successful in the prediction of global and target dose distribution, but is currently limited in its ability to predict (Dmax) values for small, elongated, or serial OAR, such as the Spinal Cord. This is because these endpoints are highly dependent on the absolute values of a few voxels, leading to extreme sensitivity to slight changes in their predictions. The next iteration of this architecture would add “patch-based” local attention mechanisms focused on small serial OARs [55], or incorporate clinical protocols directly into the loss function to strictly penalise violations of the maximum dose [56].

#### 4.4.3. Absence of Ablation Study and Component Validation

The current study does not include a formal ablation experiment isolating the contribution of each ECLoss component and architectural modification. This is acknowledged as a methodological limitation. We provide three complementary justifications for the design choices. First, each component is theoretically motivated and grounded in established literature: SharpLoss-type perceptual terms address the well-documented posterior-averaging smoothing problem in voxel-wise objectives [46]; DVH-aware losses have been formally validated in prior ablation studies, Nguyen et al. [39], who demonstrated statistically significant PTV coverage improvement versus L1 alone; gradient regularisation follows total-variation regularisation principles [36] and residual connections are supported [57]; and attention gating has been ablated and validated [35,58]. Second, our contribution is the novel joint combination and dynamic weighting of these components within a single unified ECLoss objective—rather than the introduction of individually unproven building blocks. Third, a structured ablation study is planned as a dedicated follow-up investigation. The planned experimental variants are as follows: (1) Baseline: L1 loss only, standard UNet3D without attention or residual connections; (2) SharpLoss only; (3) DVH Loss only; (4) Gradient Regularisation only; (5) Attention modules only; (6) Full ECLoss without attention modules; (7) Full proposed model (ECLoss + residual connections + attention). Each variant will be trained under identical conditions and evaluated on the same held-out test set using MAE, SSIM, and DVH-based metrics. These results will be reported in a subsequent publication.

Additionally, the model was trained and evaluated on a static planning CT basis without explicit modelling of respiratory motion. All training cases incorporated institutional motion management strategies (ITV-based planning or breath-hold techniques) in the ground-truth dose distributions. The absence of explicit 4D-CT-based motion modelling is a recognised constraint for lung radiotherapy prediction, and this will be addressed in future work through 4D dose-accumulation approaches.

#### 4.4.4. Future Directions

Moving towards creating a full treatment generation pipeline using autonomous AI methods requires closing the gap between predicting doses and delivering them with a machine. Work on future projects will focus on developing site-agnostic architectures using Federated Learning or shared institutional data to evaluate the model’s ability to generalise across different clinical sites. Embedding these models within an automated treatment planning process, where the AI-predicted dose is sent directly to a dose-mimicking optimiser, will also be critical. This approach will enable the direct auto-generation of the machine’s delivery parameters (such as Multi-Leaf Collimator (MLC) leaf sequence and MU weightings), thereby shifting from static dose predictions to autonomous treatment planning [35,59,60,61]. Additionally, incorporating uncertainty quantification for QA purposes [35,62], including explainability, will be important to ensure patient safety prior to initiating prospective clinical studies.

## 5. Conclusions

This research used an enhanced version of the UNet3D model (Enhanced UNet3D) that utilises a composite loss called Enhanced Combined Loss (ECLoss) to estimate three dimensional dose distributions for VMAT radiotherapy in patients diagnosed with lung cancer. By combining sharpness loss, target region attention loss, and three-dimensional gradient regularisation with a loss based on dose volume histograms (DVH), the developed model predicts dose maps that are consistent with those created by radiation oncologists using the clinical plan for each patient (voxel- wise, structural, and dosimetric). The enhanced UNet3D model has also been shown to produce accurate dose predictions, with a mean absolute error (MAE) of 0.238 ± 0.075 Gy and a structural similarity index (SSIM) of 0.970 ± 0.005. Due to its near-real-time inference speed of approximately 0.5 s per patient, this method can be feasibly integrated into clinical workflows for rapid plan evaluation and quality assurance. Although this method has shown positive results, several limitations remain. One limitation is that this model may not perform well if applied to data collected at other institutions with different dose trade-off preferences or a different technology platform than the one used to create the original training dataset. Additionally, accurately predicting the maximum dose (Dmax) endpoints for small or serial structures (i.e., spinal cord) remains difficult, since these metrics are very sensitive to minor voxel-wise variations in predicted doses. The following three key areas will be targeted in future research: Multi-Institutional Validation: Utilising federated learning to design site-agnostic architectures. Architectural Refining: Using both patch-based local attention and protocol-aware loss functions to enhance the model’s ability to predict (Dmax) values for organs-at-risk (OARs). Autonomous Planning: Passing the AI-generated dose maps to a dose-mimicking optimiser to generate the machine parameter files that would result in the delivery of the dose map. Safety & Quality Assurance: Developing methods to quantify the model’s uncertainty so that patient safety can be assured during prospective clinical evaluations.

## Figures and Tables

**Table 1:** Summary of patient and treatment characteristics for all test cases (*n* = 14).

Case ID	Number of PTVs	Age (Years)	Tumour Volume (cc)	Prescription Dose (Gy)	Fractionation (fr)	Lung (L/R)	Total Lung Volume (cc)
157	1	65	203.63	45	10	R	1937.16
158	1	61	163.88	60	30	L	1015.37
159	2	65	131.27	42	15	R	2304.99
160	3	80	248.3	60	30	R	4170.16
161	1	81	106.02	66	33	R	1613.21
162	4	78	327.29	60	30	L	3828.61
163	2	60	1168.16	60	30	R	3283.46
164	2	91	26.61	60	30	R	2005.72
165	4	54	531.35	66	25	L	4305.5
166	3	53	668.38	39	13	R	2154.92
167	3	52	441.4	40	10	L	2384.77
168	2	56	630.86	50	20	R	2248.59
169	2	65	99.64	39	13	R	2847.15
170	7	44	486.2	60	30	L	2245.38

**Table 2:** Summary statistics for absolute and relative dose predictions.

Metric type	Region	MAE (Mean ± SD)	SSIM (Mean ± SD)	PSNR (Mean ± SD)
Absolute dose (Gy)	All voxels †	0.238 ± 0.0752 Gy	0.970 ± 0.00570	38.10 ± 2.32 dB
	Non-zero ††	3.94 ± 0.850 Gy	0.565 ± 0.131	25.73 ± 1.96 dB
Relative dose (0–1)	All voxels	0.00460 ± 0.00129	0.960 ± 0.00790	32.18 ± 1.77 dB
	Non-zero †	0.0774 ± 0.0160	0.465 ± 0.141	19.82 ± 1.74 dB

† All voxels: refers to all voxels included in the analysis; †† Non-zero: denotes only those voxels with a dose greater than zero in the matrix.

**Table 3:** Mean absolute error (MAE) for DVH metrics (mean ± SD of |prediction − GT|) by structure and metric. *n* = number of samples (PTV: total PTVs across patients; OAR: number of patients with that structure). DVH: dose–volume histogram.

Structure	Metric	MAE Mean ± SD	*n*
Treatment Targets			
PTV	D99 (% of D_pre_)	8.871 ± 10.771	37
PTV	D98 (% of D_pre_)	8.035 ± 8.385	37
PTV	D95 (% of D_pre_)	6.227 ± 4.397	37
PTV	D5 (% of D_pre_)	6.124 ± 6.225	37
OAR			
Oesophagus	D2 (% of D_pre_)	4.335 ± 3.783	9
Oesophagus	V40 (% of volume)	2.784 ± 4.745	9
Oesophagus	V50 (% of volume)	5.604 ± 11.951	9
Heart	V35 (% of volume)	4.257 ± 5.300	14
Spinal cord	D2 (% of D_pre_)	6.662 ± 5.406	14
Lungs	D_mean (% of D_pre_)	3.306 ± 2.288	14
Lungs	V5 (% of volume)	9.459 ± 7.766	14
Lungs	V20 (% of volume)	2.641 ± 2.787	14

## Data Availability

The data presented in this study are available on request due to privacy and institutional restrictions, as this clinical dataset (retrospective VMAT lung cancer treatment plans) was sourced from the Oncology Centre at St. Teresa’s Hospital (HKSAR) and used with their explicit permission for this current study. Due to the sensitive nature of patient medical records and institutional data ownership, these data are not publicly available. Instead, data are available upon reasonable request to the corresponding author at wing.chi.chan@polyu.edu.hk. The corresponding author will evaluate the request and facilitate the necessary permissions from the Oncology Centre, St. Teresa’s Hospital (HKSAR) before any data sharing.

## Supplementary Materials

The following supporting information can be downloaded at: https://www.mdpi.com/article/10.3390/bioengineering13050490/s1, Table S1. Summary of hardware specifications, architectural configurations, and algorithmic hyperparameters for the Enhanced UNet3D and ECLoss training framework to ensure full reproducibility.
