# Supplementary material for: Deep-Learning-Based 3D Dose Distribution Prediction for VMAT Lung Cancer Treatment Using an Enhanced UNet3D Architecture with Composite Loss Functions
Source: Bioengineering (Basel). 2026 Apr 23;13(5):490. doi: 10.3390/bioengineering13050490 (PMC13203807; doi:10.3390/bioengineering13050490)
Supplement: Supplementary file 1 [file bioengineering-13-00490-s001.zip › bioengineering-4201293-supplementary.pdf]

Table S1. Summary of hardware specifications, architectural configurations, and algorithmic hyperparameters for the Enhanced UNet3D and ECLoss training framework to ensure full reproducibility.

Category	Parameter / Component	Value / Description
Hardware & Software	Hardware	Single NVIDIA GeForce RTX 4090 GPU (24 GB VRAM)
	Framework	PyTorch (version ≥ 1.4 for gradient checkpointing)
Network Architecture	Base architecture	Symmetric 3D U-Net (4 resolution levels)
	Input tensor	4-channel 3D volume (CT, PTV, OARs, Rx)
	Output tensor	1-channel 3D volume (predicted absolute dose)
	Encoder blocks	3D residual blocks (dual 3 × 3 × 3 Conv → GN → ReLU) + identity shortcut
Network Architecture	Decoder upsampling	3D transposed convolutions (kernel size 2, stride 2)
	Skip connections	Integrated Attention Gate (IAG) via element-wise addition and Sigmoid activation prior to concatenation
	Final layer activation	Linear activation function (to support unbound values > 1.0)
	Memory optimisation	Gradient checkpointing applied to selected blocks
Training Hyperparameters	Optimiser	AdamW
	Max epochs	200
	Batch size	2 (micro-batch size with gradient accumulation)
	Weight decay	3×10^{-4}
	Learning rate schedule	Warmup-cosine scheduler
	Initial learning rate	1.5×10^{-4}
	Peak learning rate	2×10^{-4} (epoch 15)
	Minimum learning rate	2×10^{-5}
	Early stopping criteria	Patience of 35 epochs without validation loss improvement
	Clinical safeguard	Halt training if average validation D_{95} error > 0.1
ECLoss	L1 regularisation (δ)	0.05 (fixed throughout training)
	Initial weights	$\alpha = 0.15$ (SharpLoss), $\beta = 0.70$ (DVH), $\gamma = 0.15$ (Gradient), $\delta = 0.05$
	Phase 1 clamp bounds	$\alpha \in [0.15, 0.35]$, $\beta \in [0.40, 0.70]$, $\gamma \in [0.10, 0.30]$
	Phase 2 clamp bounds	$\alpha \in [0.10, 0.25]$, $\beta \in [0.60, 0.75]$, $\gamma \in [0.10, 0.20]$
	Phase 2 update step sizes	$\Delta\alpha = -0.02$, $\Delta\beta = -0.025$, $\Delta\gamma = -0.015$
	SharpLoss tuning	$\gamma_{focal} = 150$; $D_{th} = 0.03$

DVH loss tuning	Sigmoid temperature parameter $\tau = 0.05$
